# Digital access constraints predict worse mental health among adolescents during COVID-19

**DOI:** 10.1038/s41598-022-23899-y

**Published:** 2022-11-09

**Authors:** Thomas E. Metherell, Sakshi Ghai, Ethan M. McCormick, Tamsin J. Ford, Amy Orben

**Affiliations:** 1 Department of Psychology, University of Cambridge, Cambridge, UK; 2 MRC Cognition and Brain Sciences Unit, 15 Chaucer Road, Cambridge, CB2 7EF UK; 3 Donders Institute, Radboud University Medical Center, Nijmegen, The Netherlands; 4 Department of Psychiatry, University of Cambridge, Cambridge, UK; 5 Present Address: Division of Psychology and Language Sciences, University College London, London, UK; 6 Present Address: Methodology and Statistics Department, Institute of Psychology, Leiden University, Leiden, The Netherlands

**Keywords:** Psychology, Risk factors

## Abstract

The COVID-19 pandemic and ensuing social restrictions disrupted young people’s social interactions and resulted in several periods during which school closures necessitated online learning. We hypothesised that digitally excluded young people would demonstrate greater deterioration in their mental health than their digitally connected peers during this time. We analysed representative mental health data from a sample of UK 10–15-year-olds (N = 1387) who completed a mental health inventory in 2017–2019 and thrice during the pandemic (July 2020, November 2020 and March 2021). We employed longitudinal modelling to describe trajectories of adolescent mental health for participants with and without access to a computer or a good internet connection for schoolwork. Adolescent mental health symptoms rose early in the COVID-19 pandemic, with the highest mean Total Difficulties score around December 2020. The worsening and subsequent recovery of mental health during the pandemic was greatly pronounced among those without access to a computer, although we did not find evidence for a similar effect among those without a good internet connection. We conclude that lack of access to a computer is a tractable risk factor that likely compounds other adversities facing children and young people during periods of social isolation or educational disruption.

## Introduction

Since the onset of the COVID-19 pandemic, populations around the world have experienced a noted decrease in mental health, with evidence for rising levels of anxiety, depression and psychological distresses^1^. Large-scale disruptions to work, education, leisure, and social activities, as well as additional pandemic stresses and healthcare problems, make such a development unsurprising. Nevertheless, specific concerns have been raised about the mental health impacts of the pandemic on adolescent populations. Adolescence represents a vulnerable period for the development of mental health disorders^2^, which can have long-lasting consequences into adulthood^3,4^. The mental health of children and adolescents in the United Kingdom was already deteriorating before the pandemic, highlighted by increases in anxiety, depression and self-harm^5–7^. Since the onset of the pandemic, however, the incidence of probable mental health conditions in this age group has risen further from 10.8% in 2017 to 16% in July 2020^8^, a trend mirrored in other studies that found deteriorating mental health in adolescents both in the UK^9^ and internationally^10^.

One of the most prominent disruptions to adolescent life during the COVID-19 pandemic has been the closure of schools and the increase in online schooling^11,12^. While school closures caused educational disruptions experienced by most adolescents^13^, their impact was not felt equally. For those adolescents who were digitally excluded, for example through lacking access to a computer or internet connection needed to successfully partake in online education, educational disruptions were much greater^14^. For example, in a UK sample, 30% of school students from middle-class homes reported taking part in live or recorded school lessons daily, while only 16% of students from working-class homes reported doing so^14^. Prior research has shown that educational disruption can negatively impact adolescent mental health^15^, and this may have resulted in negative impacts on adolescents’ mental health outcomes during the pandemic. In particular, school routines can serve as coping mechanisms in the face of mental health issues in adolescents, and therefore disruption of these routines may negatively impact adolescent mental health^16^. The closure of schools may also obstruct access to mental health support, particularly among socioeconomically disadvantaged students and ethnic minorities^17^.

COVID-19 and the subsequent lockdown measures (including school closures) also brought with them a curtailing of general social contact and widespread social disruption. At times when in-person peer interaction was cut to a minimum, online and digital forms of interaction with peers (e.g., through online schooling or social media) might have helped buffer some of these social disruptions^18,19^. Lack of access to the technologies necessary to support online interactions in a schooling context could have led to negative mental health consequences, especially as adolescent cognitive, biological and social development makes them more sensitive to limitations in social contact and decreases in peer interaction^20^. We therefore examined whether lack of access to the computer or good internet connection needed for school had the potential to further exacerbate mental health impacts given technology’s crucial role in enabling adolescents to participate in both educational and social activities.

In this study, we use Understanding Society, a large longitudinal panel survey from the United Kingdom to test whether the mental health trajectories of adolescents who were digitally excluded during the pandemic differed from those of their digitally included counterparts. Digital exclusion is a multifaceted problem^21,22^ which can be defined as experiencing connectivity and accessibility barriers, as well as lacking digital skills and motivation^23^. With digital literacy mandated for children at UK primary schools^24^ and motivation to digitally connect in adolescence being high^25^, our study focused on the impact of lack of connectivity and accessibility. Specifically, we examined the result of not having access to a computer or good internet connection for schoolwork. While the question of how such digital exclusion affected adolescents during the pandemic has not been systematically investigated, research has found that adolescent mental health was not uniformly impacted during this time^26^, and that more socioeconomically disadvantaged children and young people showed worse mental health^27^. We therefore first examine trends in mental health across the pandemic using latent growth curve modelling, and then test whether these models differ for those without access to a computer or good internet connection compared with those with this digital access.

## Results

Before fitting our longitudinal models, we cleaned our data and excluded participants without a longitudinal weight or any mental health scores. 1388 participants had an assigned longitudinal weight^28^ for COVID-19 wave 8, of which 1 (0.07%) had no Total Difficulties scores and so was excluded, leaving 1387 (656 [47.3%] male, 731 [52.7%] female) adolescents to be included in our analyses. Of these, 638 (46.0%) had a Total Difficulties score in main study wave 9, 818 (59.0%) in COVID-19 wave 4, 836 (60.3%) in COVID-19 wave 6 and 1386 (99.9%) in COVID-19 wave 8. Full characteristics of participants with missing responses are provided in Supplementary Table S1. Of the 1387 included participants, 836 (60.3%) had a valid response to the digital inclusion question. Table 1 shows the sociodemographic characteristics of participants in relation to their responses to the digital inclusion question.

**Table 1:** Digital inclusion characteristics according to key sociodemographic variables (ethnicity data suppressed due to low numbers to protect the identities of participants).

Group	Response type			Has a computer/has access to a good internet connection	
	All	Available	Unavailable	Yes/Yes	At least one no
Total	1387	836	551	714	122
**Sex**					
Male	656	386	270	343	43
Female	731	450	281	371	79
Unavailable	0	0	0	0	0
**Birth year**					
2004–2007	781	481	300	419	62
2008–2011	606	355	251	295	60
Unavailable	0	0	0	0	0
**Ethnicity**					
White	1005				
Ethnic minority	367				
Unavailable	15				
**Mean household income (x, annual)**					
x < £40,000	378	250	128	205	45
£40,000 ≤ x	407	260	147	228	32
Unavailable	602	326	276	281	45

In the sensitivity check (see “Methods”), we also included those participants without a longitudinal weight (see Supplementary Tables S12–15 and Figs. S1, 2), incorporating a total of 1422 valid responses to the digital inclusion question.

### Overall profile of mental health

**Figure 1:**
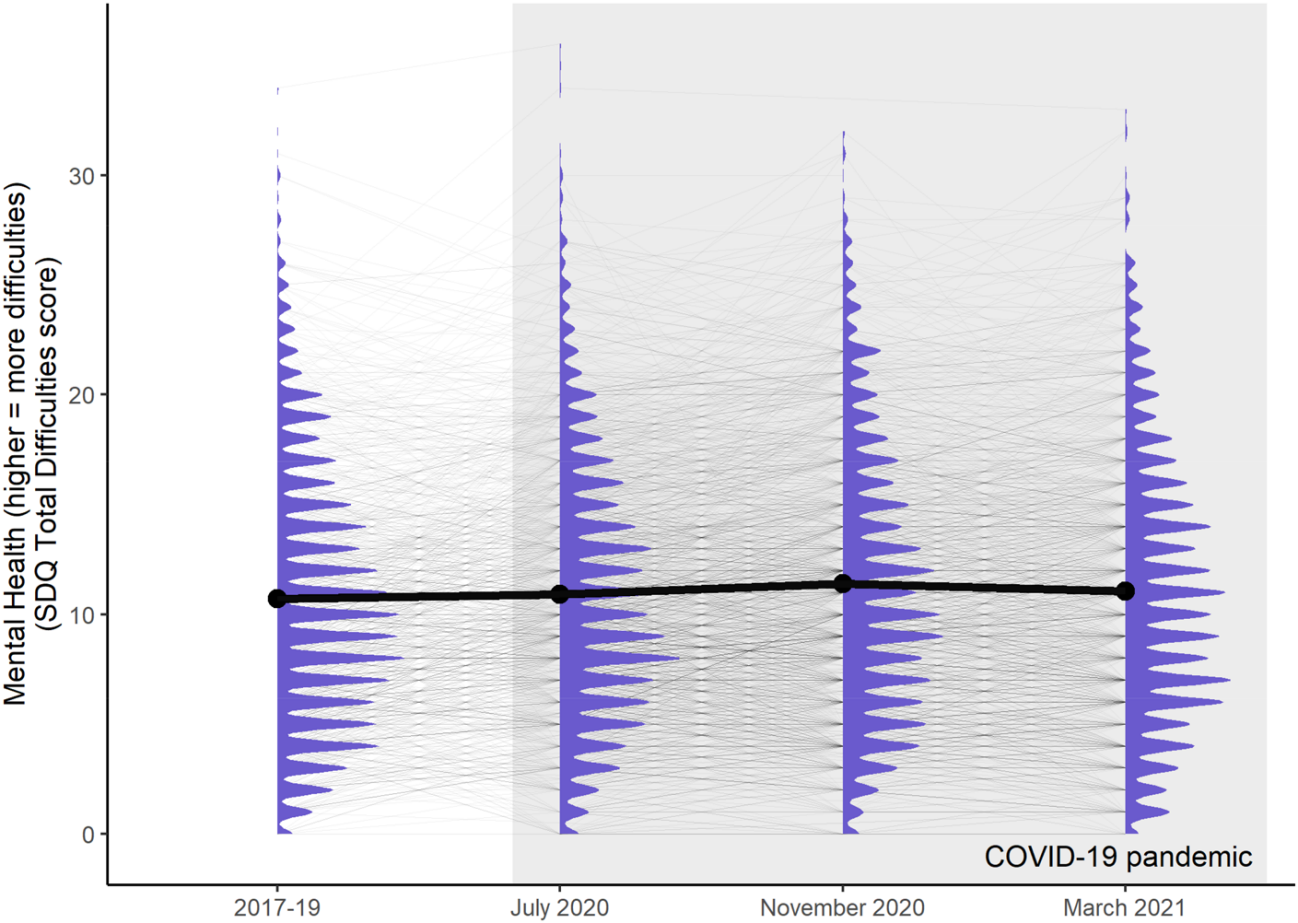
Adolescent SDQ Total Difficulties scores, 2017 to March 2021. For each wave, we plotted the distribution of mental health scores (blue violin plots). The mean of each wave (black) is included to highlight the change in average scores between waves, and individual raw scores are also displayed (grey). The timing of the COVID-19 pandemic is indicated in grey.

To understand the general trend in mental health over time, we first examined the raw SDQ data. The mean Total Difficulties score was 10.7 (SD 5.93; out of a maximum 40) in main study wave 9 (2017–2019), rose through COVID-19 wave 4 with a value of 10.9 (SD 6.02) and peaked at 11.4 (SD 6.33) in COVID-19 wave 6, before declining to 11.1 (SD 6.14) in COVID-19 wave 8 (Fig. 1). This shows that there are small changes in mental health across the pandemic.

### Establishing developmental trajectories

**Figure 2:**
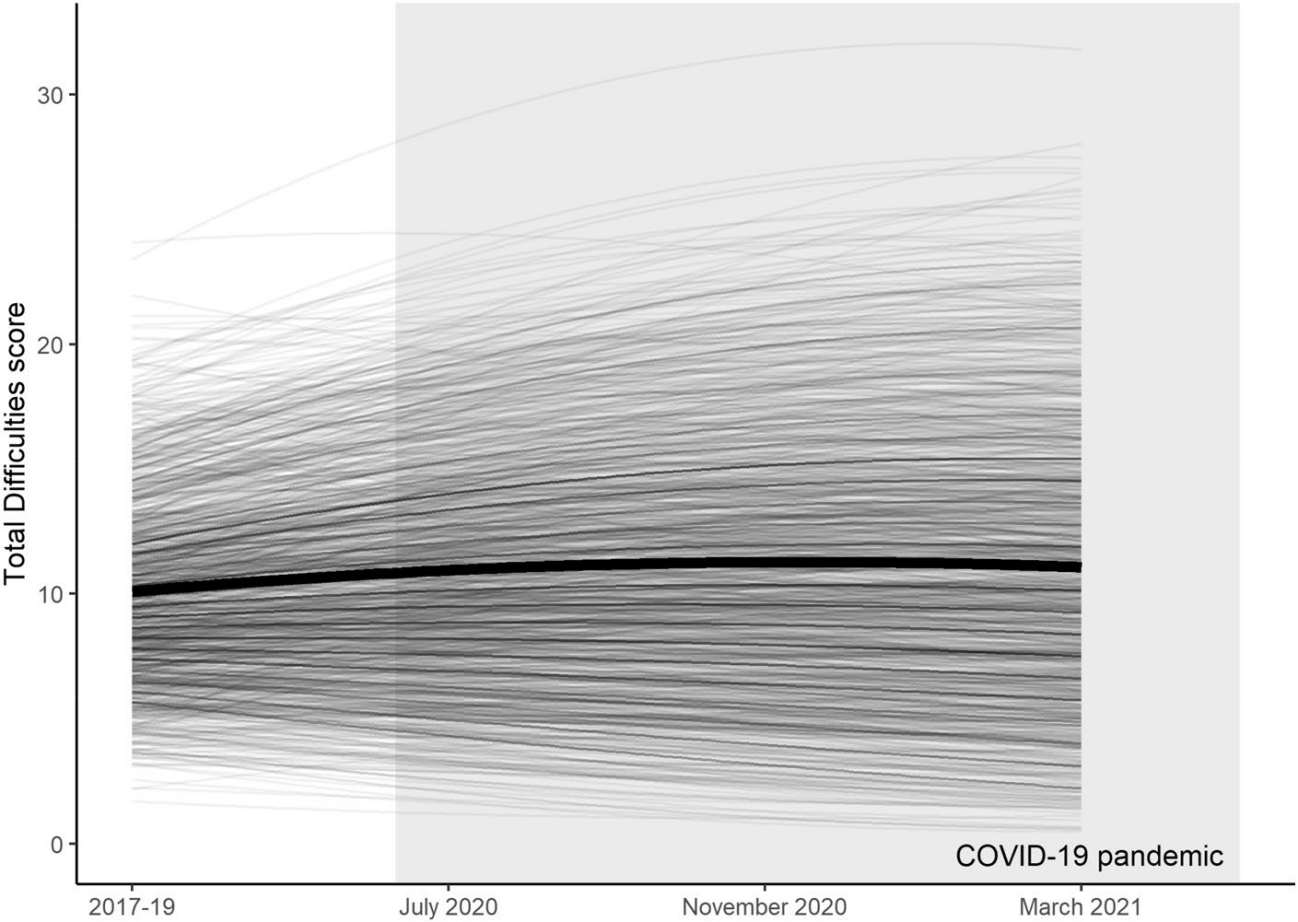
Latent growth curve model (bold) of adolescent SDQ Total Difficulties scores between 2017 and March 2021, based on the Understanding Society dataset. Individual predicted trajectories are also shown, along with the timing of the COVID-19 pandemic in grey.

To find a best-fit ungrouped latent growth curve model for the whole adolescent dataset, we initially fit an intercept-only model to the whole-cohort Total Difficulties score data (${\chi }^{2}$ = 182, BIC = 22,133) and compared it to a linear model (${\chi }^{2}$ = 34.7, BIC = 22,008; LRT *p* < 2 × 10^–16^), which in turn was compared to a quadratic model (${\chi }^{2}$ = 0.157, BIC = 22,002; LRT *p* = 6 × 10^–7^; for full model fit details see Supplementary Tables S2, 3). Based on the significant improvement in model fit, we concluded that the quadratic model (Fig. 2) was most appropriate for these data. This supports the previous raw data (Fig. 1) showing that mental health followed a quadratic trajectory through the COVID-19 pandemic.

### Digital inclusion and mental health trajectories

To investigate the impact of computer and internet access on the mental health trajectories plotted in Fig. 2, we then fit multi-group latent growth curve models where parameters of interest were selectively constrained to be equal or allowed to vary between digitally excluded and included groups. We fit such multi-group models for both access to a computer and a good internet connection separately.

In each case, we have provided one model without control variables, but with survey weights applied; and one model with control variables, but without survey weights applied. This is because attempts to fit models with both control variables and survey weights applied returned errors in *lavaan*, and therefore we could not produce a generalisable model that incorporates survey weights.

#### Access to a computer

**Figure 3:**
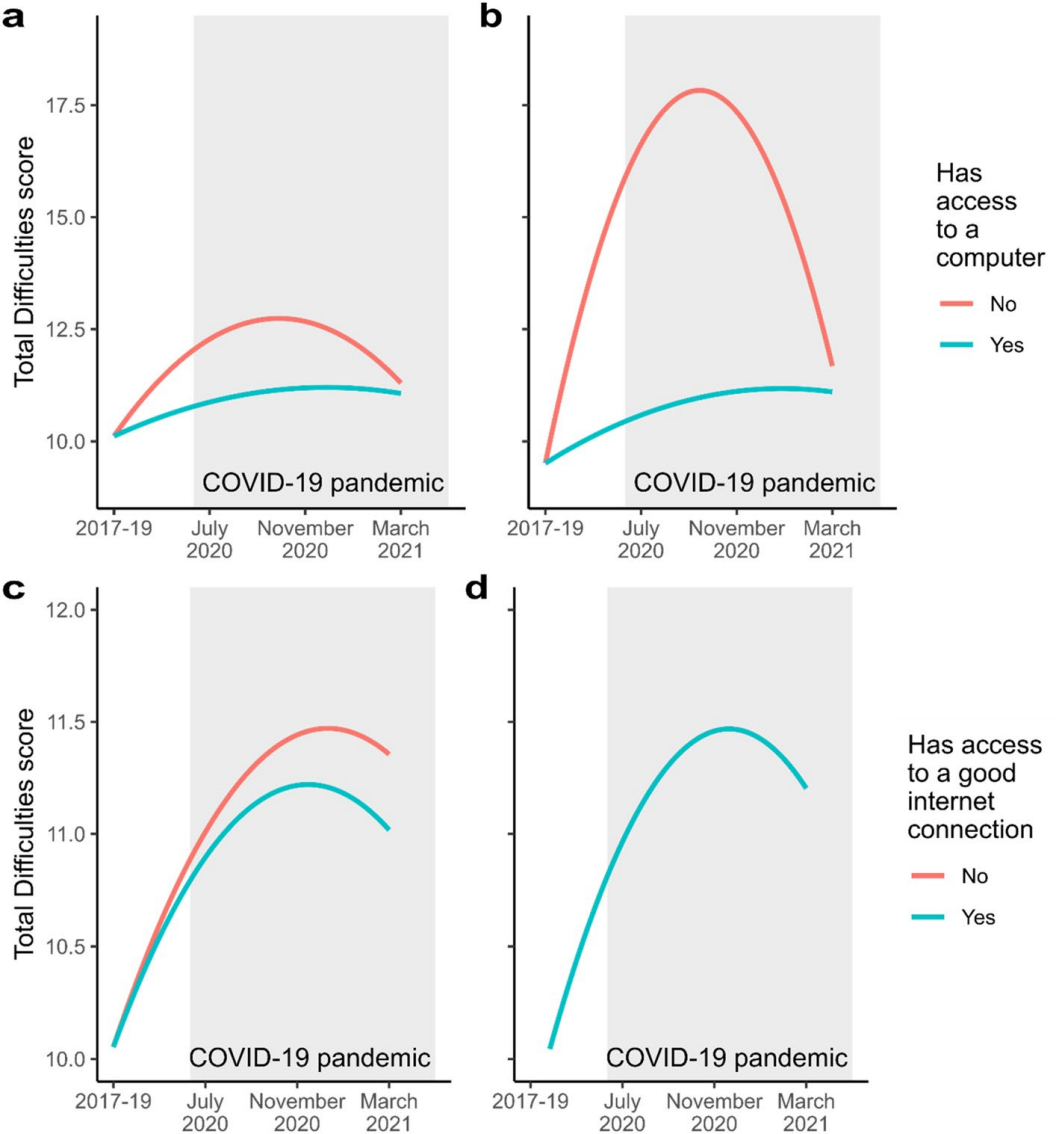
Latent growth curve models of youth SDQ Total Difficulties scores, grouped by each of the two digital inclusion criteria. (**a**) and (**b**) show the models grouped by access to a computer—(**a**) does not include sociodemographic control variables, but does have survey weights applied, while (**b**) does include the control variables but does not have weights applied (since this process is not robust given the small size of the digitally excluded group). The same apply for (**c**) and (**d**), which portray the models grouped by access to a good internet connection—in the latter, the modelled trajectories are identical.

For computer access, we found that the modelled linear and quadratic coefficients differed between groups in both models without sociodemographic variables added as control variables (LRT *p* = 0.006; *p* = 0.004 respectively; for full details see Supplementary Tables S4, 5) and models where control variables were included (LRT *p* = 7 × 10^–4^; *p* = 0.004 respectively; for full details see Supplementary Tables S6, 7). The group with no computer access has a greatly pronounced increase in mental health symptoms in the early stages of the pandemic, but these returned almost to the level of the group with computer access by COVID-19 wave 8, with (Fig. 3b) or without (Fig. 3a) taking sociodemographic variables into account.

We additionally used our final model parameters to predict individual Total Difficulties score trajectories through the period of interest. We compared the maximum score attained in each trajectory against standard clinical cut-offs^29^. In the group without computer access, 12/51 (24%) entered the “high” or “very high” range of Total Difficulties (i.e. reached a maximum score of 18 of higher), and 8/51 (16%) entered the “very high” range (maximum score of 20 or higher). In the group with computer access, these figures were 110/785 (14%) and 63/785 (8%) respectively.

It is worth noting that, although the modelled value in the 2017–2019 wave is the same for both groups, in the raw data the mean Total Difficulties score for those without computer access is 11.2 (standard error 0.89), while for those with computer access it is 9.82 (SE 0.24).

#### Access to a good internet connection

We applied the same process to test whether access to a good internet connection was associated with a change in mental health trajectory across COVID-19. Without accounting for sociodemographic variables (Fig. 3c), the group without good internet access appears to have a slightly more pronounced trajectory, with linear coefficients differing between groups (LRT *p* = 0.034; for full details see Supplementary Tables S8, 9), however this effect is not significant with control variables accounted for (Fig. 3d; see also Supplementary Tables S10, 11), eliminating trajectory differences between groups.

**Figure 4:**
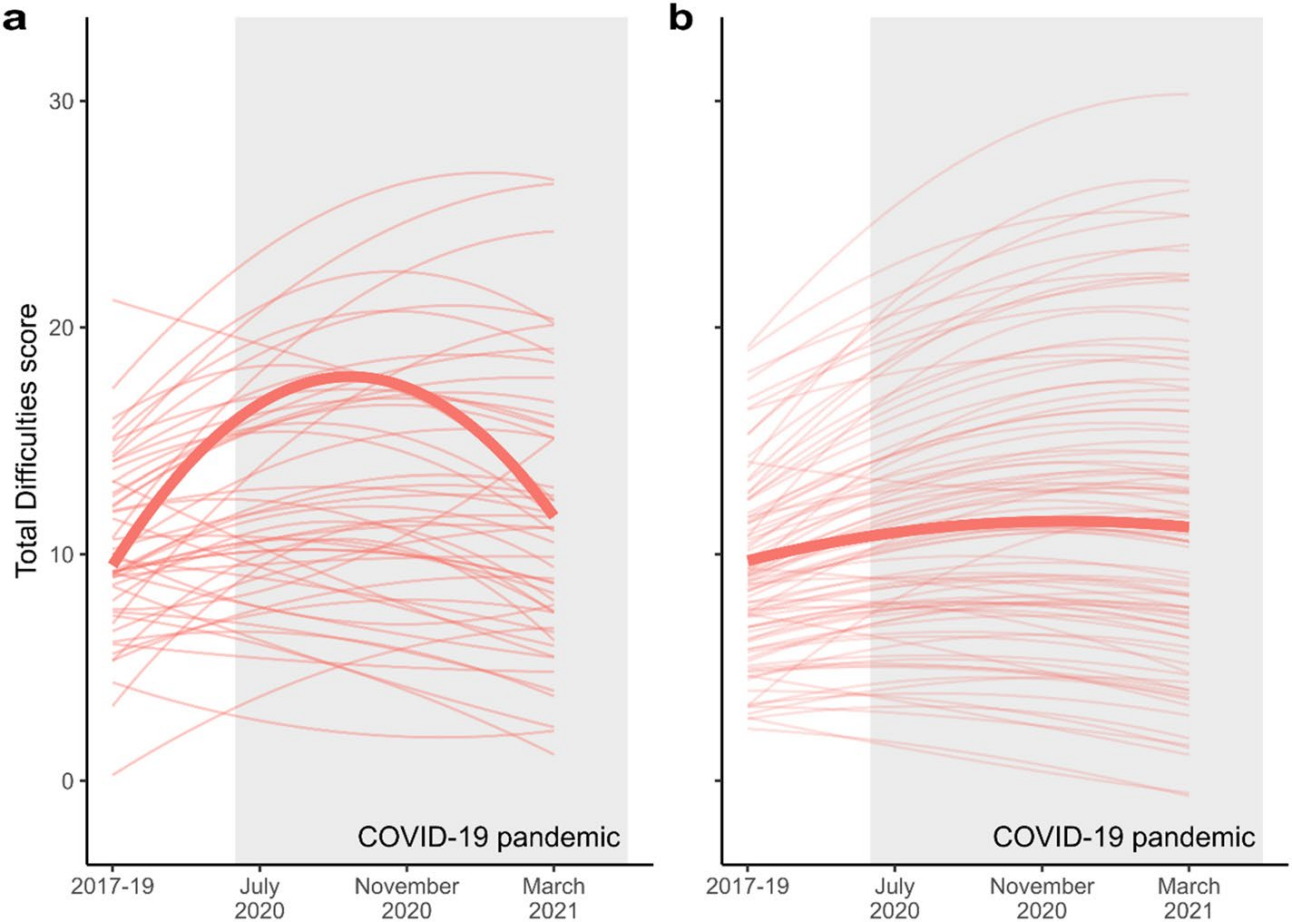
Latent growth curve models of SDQ Total Difficulties scores for those adolescents without access to a computer (**a**) and without access to a good internet connection (**b**). Predicted individual trajectories are also shown and demonstrate the size of the group in each case.

Figure 4 shows the modelled trajectories for the groups without access to a computer and without access to a good internet connection, along with predicted individual trajectories. What is evident in the figure is the small size of the digitally excluded group both for computer access (51 participants) and access to a good internet connection (90 participants).

### Sensitivity check

To confirm that excluding those participants without assigned longitudinal sample weights (see “Methods”) does not substantially affect the results of our analysis, we conducted a sensitivity check including both those with and those without longitudinal weights. These whole-cohort models had only one difference, in that in the “good internet connection” case, the difference in linear coefficients between the groups with and without access became significant (LRT *p* = 0.042). Full details of these analyses can be found in Supplementary Tables S12–15 and Figs. S1, 2.

## Discussion

In this study, we tested whether adolescent longitudinal mental health trajectories during the COVID-19 pandemic were different for adolescents who had experienced a lack of access to a computer or good internet connection during that time to those who had not. When examining all the participating adolescents, we found a small quadratic trend in mental health: symptoms increased from pre-pandemic baselines in 2020 and then decreased in early 2021. The trajectories were more pronounced for those who did not have access to a computer for online schooling, showing a greater increase during 2020 and then a greater decrease in early 2021. In contrast, we found no significant difference between the trajectories for those who had and did not have access to a good internet connection.

To understand these results, it is important to track how the educational and social disruptions experienced by UK adolescents differed across our study waves. In March 2020, UK schools were shut due to the COVID-19 pandemic, except for children of key workers or those who were considered vulnerable to lack of support from school. Furthermore, attendance among these groups was much lower than predicted^13^. While some schools did reopen before the 2020 summer holidays, attendance was not compulsory and often part-time, especially for older pupils in secondary schools. A full reopening of UK schools only occurred at the beginning of the new school year in September 2020. November 2020 saw a wave of localised restrictions, following which schools were closed again nationally in December 2020 and remained closed until March 2021. Like the Co-Space study, we found mental health symptoms to be worst during times of high COVID-19 restrictions, which would have caused both educational and social disruptions, while mental health recovered to some extent with schools reopening and social restrictions lifting in March 2021^30^.

Our analyses highlighted that those who did not have access to a computer had worse mental health during times of school closures and social isolation than those who did, which echoes the findings of the English Mental Health of Children and Young People survey follow-ups in 2020 and 2021^27,31^. Our results were robust even when controlling for sex, age, ethnicity, and household income, which is important as digital exclusion is more likely to co-occur with other adversities. Indeed, both English surveys’ follow-ups demonstrated clustering of various impairments to accessing online schooling in addition to access to a device, including a quiet space to study, support from parents and school and access to other learning resources^27,31^. A possible explanation of our results is that digitally excluded adolescents experienced much greater educational and social disruption: a lack of computer access may preclude consistent and active engagement in online schooling and keeping in touch with peers online. These adolescents did not have an effective way to buffer the lack of education or in-person social contact when lockdown measures curtailed their ability to go to school or meet face-to-face, and not having access to such devices can therefore be related to decreased mental health.

There was a clear negative relationship between not having access to a computer for school and mental health, but no evidence for a similar association in terms of not having access to a “good internet connection”. There are multiple possible explanations for this finding. First, the disruption due to not having a good internet connection (as opposed to having no internet access at all) might not be as severe as not having access to a computer, and the experiences of an adolescent might not have been that different to their peers with a good internet connection. Second, what counts as a “good” internet connection could differ across participants and therefore the measure might have been noisy. This also applies to a lesser extent to computer access, as some participants may have interpreted the term “computer” as including tablets, smartphones or other devices. As this study highlights the need for further research and policy discussion about digital exclusion in light of the COVID-19 pandemic, care needs to be taken to understand the long-term impact of digital exclusion^32^. Future research should employ a mixed methods approach to unpick the lockdown experiences of young people and elucidate which aspects of the disruption were most difficult to cope with. In particular, it should take a qualitative approach to assessing the extent and manner of digital exclusion that young people are facing, and the relationship this has with their mental health during times of social isolation. It should also consider mediation analyses as a way to pin down the mechanisms through which a lack of digital access may impact mental health. It is further possible that the relationship we have identified may extend to other life events beyond the pandemic—therefore, future work may also wish to examine the mental health impacts of digital exclusion beyond the pandemic. It is important to consider these potential effects as digital exclusion frequently co-occurs with other socio-economic disadvantages^33,34^, with a possibility of associated inequity in mental health risk.

In all, there is a lack of high-quality longitudinal data on digital exclusion and its potential impacts, and further research is necessary. However, our findings combined with those of others^30,31^ suggest that ensuring access to a computer or tablet may be a simple but important intervention should further school closures become necessary.

Our study is limited by the unequal spacing between the four waves in our longitudinal models. In addition, severely digitally excluded adolescents are in the minority in the UK, and therefore the numbers of adolescent participants in our digitally excluded groups were relatively low. Future research should over-sample diverse, digitally excluded populations, especially from rural areas within the UK, in order to achieve a larger sample from these groups. As missing data in the grouping variables could not be imputed, the statistical power of our multigroup latent growth curve model analysis was somewhat limited, although our sensitivity checks (see Supplementary Tables S12–15 and Figs. S1, 2) corroborate the study conclusions. Also, longitudinal sampling weights could not be applied in either case for models incorporating sociodemographic variables, meaning that, for now, those findings cannot be generalised to the whole UK population. Furthermore, the dichotomous nature of the survey items used obscures some of the more subtle variations in digital access, and may cause participants’ responses to not precisely reflect their true digital circumstances. For example, infrequent access to a public computer may not be well captured by either of the available binary responses. Finally, because of the focus on educational disruption, we have not investigated the implications of inequities in access to other technologies such as smartphones. Smartphones may be used by adolescents to access services like social media, but are less likely to be used for educational purposes. It should be noted, however, that around 65% of those adolescents who reported not having access to a computer in November 2020 did have access to a smartphone, so it would appear that having access to a smartphone was not sufficient to mitigate the impacts of not having access to a computer.

In conclusion, we provide evidence towards a negative impact of one type of digital exclusion, namely a lack of access to a computer, on adolescent mental health during COVID-19. We emphasise the urgent need for researchers, public health workers and policy professionals to consider and address digital exclusion as a predictor of adolescent mental health outcomes, especially during the COVID-19 pandemic when much of educational and social life moved onto digital spaces. With digitalisation becoming increasingly widespread in society, ever more services—whether they be educational, social or health-related—are “digital first”, excluding those with little or no access to the devices necessary to engage in these activities^35^. In the public and scientific conversation that predominantly focuses on the negative impacts of digital technologies on adolescent mental health^36^, the importance of obtaining basic levels of digital access as a way of supporting adolescent mental health needs to be emphasised more regularly and taken more seriously.

## Methods

### Study design and participants

In this study, we analysed data from the UK Household Longitudinal Study (Understanding Society)^37,38^, a longitudinal survey of around 40,000 UK households, with data collected annually from adults and adolescents since January 2009. At age 10, household members are first included in the survey via a paper self-completion ‘youth questionnaire’; participants migrate to the adult questionnaire at age 16. In this study we used youth questionnaire data from wave 9 as the baseline for our analysis (with invitations being issued between January 2017 and December 2018)—this being the last main study wave for which youth mental health data are available. During COVID-19, additional youth surveys were administered bimonthly between April and July 2020, and every four months between September 2020 and March 2021. Here we use data from COVID-19 waves 4, 6 and 8, dictated by when the mental health questionnaire was issued as part of the survey. 2862 unique youth questionnaires were returned in main study wave 9 (2017–2019), 1411 in COVID-19 wave 4 (July 2020), 1432 in COVID-19 wave 6 (November 2020) and 1388 in COVID-19 wave 8 (March 2021). All youths from the main Understanding Society samples were eligible for the COVID-19 survey, and 42.1% of households responded in the first wave. In households with more than one participant eligible for the youth survey, the survey was sent to all of them. We employed longitudinal sample weights as calculated and described by the data custodians for COVID-19 wave 8^28^ to correct for the deviation of the sample characteristics from those of the population, particularly the oversampling of ethnic minorities.

The University of Essex Ethics Committee approved all data collection for the Understanding Society main study and innovation panel waves, including asking consent for all data linkages except to health records.

### Procedures

To measure mental health, we used the Strengths & Difficulties Questionnaire (SDQ)^39^ (paper, self-completed and returned in a sealed envelope), which was included in the youth questionnaire in odd-numbered main study waves (until wave 9 in 2017–2019) and even-numbered COVID-19 waves (from wave 4 in July 2020). The SDQ comprises 25 items that assess common childhood psychological difficulties through a series of positively and negatively phrased statements, which are rated as ‘not true’, ‘somewhat true’ and ‘certainly true’. Items are subsequently scored from 0 to 2 as appropriate, so that a high score indicates greater difficulty. The SDQ has five subscales: hyperactivity/inattention, prosocial behaviour, emotional, conduct and peer relationship problems. The total of all but the prosocial scale are summed to provide a total difficulty score that ranges from 0 to 40, which we used to measure adolescent mental health^39^ in 2017–2019 (main study) and July 2020, November 2020 and March 2021 (COVID-19 survey).

To group participants by digital inclusion, we used the following question, included in the COVID-19 wave 6 (November 2020) youth questionnaire (paper, self-complete): “Which of these things do you have at home to help you do your school work?” The question included the response options “Access to a… computer” and “…good internet connection”. No additional definition of the terms “computer” or “good internet connection” was given to participants.

The sociodemographic variables used as control variables in this study were sex (male vs female), age (on 15th August 2020 in whole years), ethnicity (dichotomised, White vs ethnic minorities; these three derived from youth questionnaire responses) and household income (monthly, averaged across the four waves of interest; derived from the individual responses of adults in the same household).

### Statistical analysis

Participants with a calculated youth longitudinal weight^28^ for COVID-19 wave 8 (March 2021), and at least one recorded SDQ Total Difficulties score across the four waves, were included in this analysis (N = 1387). Missing Total Difficulties scores were imputed using full information maximum likelihood estimation. When grouping by computer or internet access, participants with missing data for the digital inclusion question (39.7%, 551 participants) were excluded (leaving n = 836). Analyses were conducted in R using the R package *lavaan*^40^ and graphs produced using the R package *ggplot2* among others. Sampling probability weights were accounted for in analyses with a sufficiently large sample using the R packages *survey*^41^ and *lavaan.survey*^42^.

We analysed the data by fitting latent growth curve models^43^ to the Total Difficulties scores, including multi-group models to model disparate mental health trajectories of adolescents with and without computer and internet access. Full information maximum likelihood estimation^44,45^ was employed to minimise the biasing impact of missing data. We took a sequential model-selection approach with two main components: (1) establishing the proper functional form of the mental health trajectory experienced by adolescents during the COVID-19 pandemic, and (2) establishing the appropriate level of measurement invariance^46^ between digitally included and excluded groups. Model comparisons were performed via pairwise scaled ${\chi }^{2}$ tests of goodness of fit^40^ (i.e. likelihood ratio test; LRT). To test for functional form, we first compared an intercept-only model to a linear model, and then a quadratic model. For the multi-group models, additional pairwise comparisons were conducted to test model fit with equality constraints between groups, including factor variances, factor covariances and the Total Difficulties residual variances. In so doing we established whether there is significant evidence for differences in model parameters between the two groups in each case. Mis-specified models (e.g., models with negative variances or a non-positive definite covariance matrix) were discarded during the model building process. To probe the robustness of our analyses, we also conducted a sensitivity check where those participants without longitudinal weights were not excluded, and refit models with sociodemographic covariates grouping by both computer and good internet connection access (see Supplementary Tables S12–15 and Figs. S1, 2).

## Supplementary Information

Supplementary Information.

## Data Availability

All data used in this study are publicly available via the UK Data Service (study numbers 6614 and 8644). R code used for analysis is available at https://osf.io/qhtbj/.

## References

[1] Xiong J (2020). Impact of COVID-19 pandemic on mental health in the general population: A systematic review. J. Affect. Disord..

[2] Costello EJ, Copeland W, Angold A (2011). Trends in psychopathology across the adolescent years: What changes when children become adolescents, and when adolescents become adults?: Trends in psychopathology across the adolescent years. J. Child Psychol. Psychiatry.

[3] Kessler RC, Petukhova M, Sampson NA, Zaslavsky AM, Wittchen H-U (2012). Twelve-month and lifetime prevalence and lifetime morbid risk of anxiety and mood disorders in the United States: Anxiety and mood disorders in the United States. Int. J. Methods Psychiatr. Res..

[4] Ormel J (2017). Functional outcomes of child and adolescent mental disorders. Current disorder most important but psychiatric history matters as well. Psychol. Med..

[5] McManus S (2019). Prevalence of non-suicidal self-harm and service contact in England, 2000–14: Repeated cross-sectional surveys of the general population. Lancet Psychiatry.

[6] Pitchforth J (2019). Mental health and well-being trends among children and young people in the UK, 1995–2014: Analysis of repeated cross-sectional national health surveys. Psychol. Med..

[7] Sellers R (2019). Cross-cohort change in adolescent outcomes for children with mental health problems. J. Child Psychol. Psychiatry.

[8] Vizard, T. *et al.**Mental Health of Children and Young People in England, 2020* https://files.digital.nhs.uk/CB/C41981/mhcyp_2020_rep.pdf (2020).

[9] Waite P (2021). How did the mental health symptoms of children and adolescents change over early lockdown during the COVID-19 pandemic in the UK?. JCPP Adv..

[10] Barendse, M. *et al. Longitudinal change in adolescent depression and anxiety symptoms from before to during the COVID-19 pandemic: A collaborative of 12 samples from 3 countries* (PsyArXiv, 2021) 10.31234/osf.io/hn7us.

[11] Bubb S, Jones M-A (2020). Learning from the COVID-19 home-schooling experience: Listening to pupils, parents/carers and teachers. Improv. Sch..

[12] Dhawan S (2020). Online learning: A panacea in the time of COVID-19 crisis. J. Educ. Technol. Syst..

[13] Cooper, K. & Stewart, K. *Does Money Affect Children’s Outcomes? An update* 1–41 https://sticerd.lse.ac.uk/CASE/_NEW/PUBLICATIONS/abstract/?index=5662 (2017).

[14] Cullinane, C. & Montacute, R. *COVID-19 and Social Mobility Impact Brief #1: School Shutdown* (2020).

[15] Viner RM (2020). School closure and management practices during coronavirus outbreaks including COVID-19: A rapid systematic review. Lancet Child Adolesc. Health.

[16] Lee J (2020). Mental health effects of school closures during COVID-19. Lancet Child Adolesc. Health.

[17] Colvin MK, Reesman J, Glen T (2022). The impact of COVID-19 related educational disruption on children and adolescents: An interim data summary and commentary on ten considerations for neuropsychological practice. Clin. Neuropsychol..

[18] Ofcom (2020). Ofcom Children’s Media Lives: Life in Lockdown.

[19] Orben A, Tomova L, Blakemore S-J (2020). The effects of social deprivation on adolescent development and mental health. Lancet Child Adolesc. Health.

[20] Blakemore S-J (2019). Adolescence and mental health. Lancet.

[21] van Deursen, A. J. A. M. & Helsper, E. J. In *Stud. Media Commun.* Vol. 10 (eds Robinson, L. *et al.*) 29–52 (Emerald Group Publishing Limited, 2015).

[22] Helsper, E. The digital disconnect: The social causes and consequences of digital inequalities. *Digit. Disconnect* 1–232 (2021).

[23] NHS Digital. What we mean by digital inclusion. https://digital.nhs.uk/about-nhs-digital/our-work/digital-inclusion/what-digital-inclusion-is (2021).

[24] Ofsted. ICT in schools: 2008 to 2011. *GOV.UK* https://www.gov.uk/government/publications/ict-in-schools-2008-to-2011 (2011).

[25] Anderson, M. & Jiang, J. 1. Teens and their experiences on social media. *Pew Res. Cent. Internet Sci. Tech* https://www.pewresearch.org/internet/2018/11/28/teens-and-their-experiences-on-social-media/ (2018).

[26] Ford T, John A, Gunnell D (2021). Mental health of children and young people during pandemic. BMJ.

[27] Newlove-Delgado T (2021). Child mental health in England before and during the COVID-19 lockdown. Lancet Psychiatry.

[28] University of Essex, Institute of Social and Economic Research. *Understanding Society COVID-19: User Guide*. https://www.understandingsociety.ac.uk/sites/default/files/downloads/documentation/covid-19/user-guides/covid-19-user-guide.pdf (2021).

[29] Goodman, R. *Scoring the Strengths & Difficulties Questionnaire for Age 4–17 or 18+* https://www.sdqinfo.org/py/sdqinfo/b3.py?language=Englishqz(UK) (2014).

[30] Raw JAL (2021). Examining changes in parent-reported child and adolescent mental health throughout the UK’s first COVID-19 national lockdown. J. Child Psychol. Psychiatry.

[31] Newlove-Delgado, T. *et al.* Mental Health of Children and Young People in England 2021—wave 2 follow up to the 2017 survey. *NHS Digit.* https://digital.nhs.uk/data-and-information/publications/statistical/mental-health-of-children-and-young-people-in-england/2021-follow-up-to-the-2017-survey (2021).

[32] Livingstone S, Helsper E (2007). Gradations in digital inclusion: Children, young people and the digital divide. New Media Soc..

[33] Watts G (2020). COVID-19 and the digital divide in the UK. Lancet Digit. Health.

[34] Livingstone S, Blum-Ross A (2020). Parenting for a Digital Future: How Hopes and Fears about Technology Shape Children’s Lives.

[35] Robotham D, Satkunanathan S, Doughty L, Wykes T (2016). Do we still have a digital divide in mental health? A five-year survey follow-up. J. Med. Internet Res..

[36] Orben A (2020). The sisyphean cycle of technology panics. Perspect. Psychol. Sci..

[37] University Of Essex, Institute For Social & Economic Research. Understanding Society: Waves 1-10, 2009-2019 and Harmonised BHPS: Waves 1–18, 1991–2009 10.5255/UKDA-SN-6614-14 (2021).

[38] University Of Essex, Institute For Social & Economic Research. Understanding Society: COVID-19 Study, 2020–2021. 10.5255/UKDA-SN-8644-10 (2021).

[39] Goodman R, Meltzer H, Bailey V (1998). The strengths and difficulties questionnaire: A pilot study on the validity of the self-report version. Eur. Child Adolesc. Psychiatry.

[40] Rosseel Y (2012). lavaan: An R package for structural equation modeling. J. Stat. Softw..

[41] Lumley, T. *survey: Analysis of Complex Survey Samples*. https://CRAN.R-project.org/package=survey (2021).

[42] Oberski D (2014). lavaan.survey: An R package for complex survey analysis of structural equation models. J. Stat. Softw..

[43] McArdle JJ (2009). Latent variable modeling of differences and changes with longitudinal data. Annu. Rev. Psychol..

[44] Enders CK (2001). The performance of the full information maximum likelihood estimator in multiple regression models with missing data. Educ. Psychol. Meas..

[45] Rosseel Y (2021). Evaluating the observed log-likelihood function in two-level structural equation modeling with missing data: From formulas to R code. Psych.

[46] Meredith W (1993). Measurement invariance, factor analysis and factorial invariance. Psychometrika.

