# Supplementary material for: Digital access constraints predict worse mental health among adolescents during COVID-19
Source: Sci Rep. 2022 Nov 9;12:19088. doi: 10.1038/s41598-022-23899-y (PMC9645341; doi:10.1038/s41598-022-23899-y)
Supplement: Supplementary file 1 — Supplementary Information. [file 41598_2022_23899_MOESM1_ESM.pdf]

Supplementary information for
Digital access constraints predict worse mental health among adolescents during COVID-19

Thomas E. Metherell, Sakshi Ghai, Ethan M. McCormick, Tamsin J. Ford, and Amy Orben*

*Correspondence should be addressed to Amy Orben
Email: amy.orben@mrc-cbu.cam.ac.uk

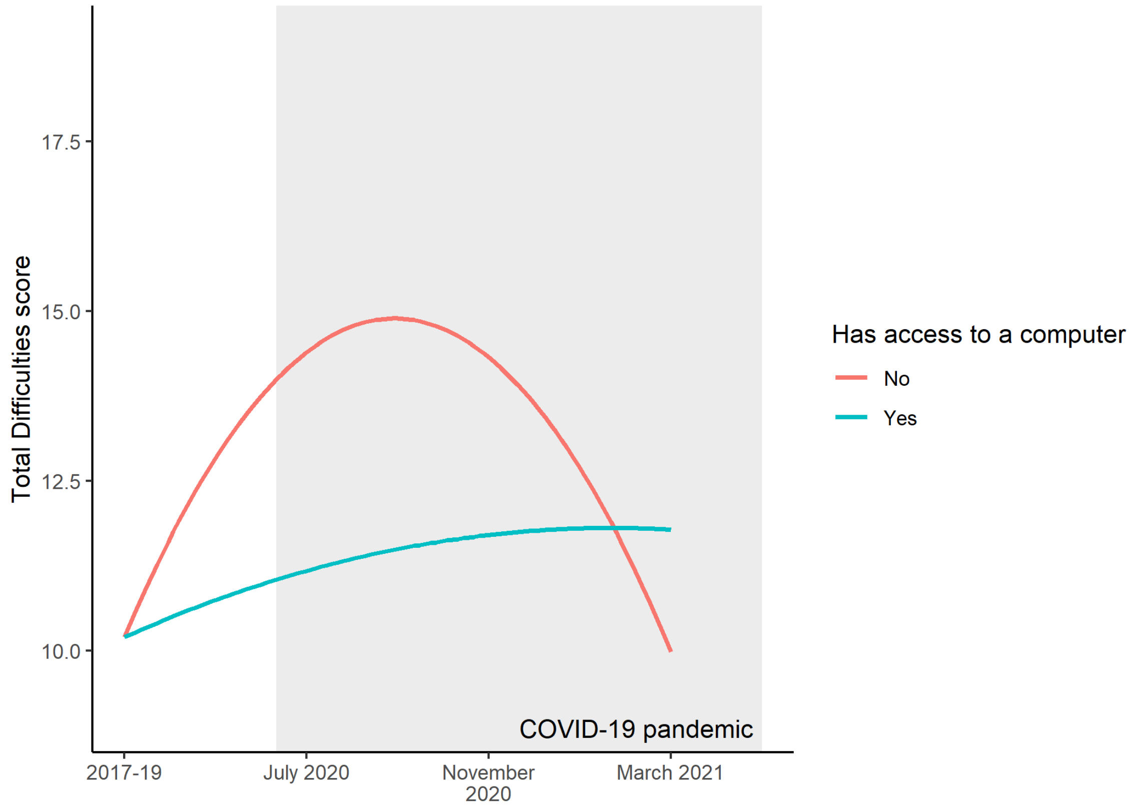

Fig. S1. Sensitivity check: latent growth curve model of youth SDQ Total Difficulties scores, grouped by computer access, including sociodemographic control variables and without excluding participants with no longitudinal weight for COVID-19 wave 8.

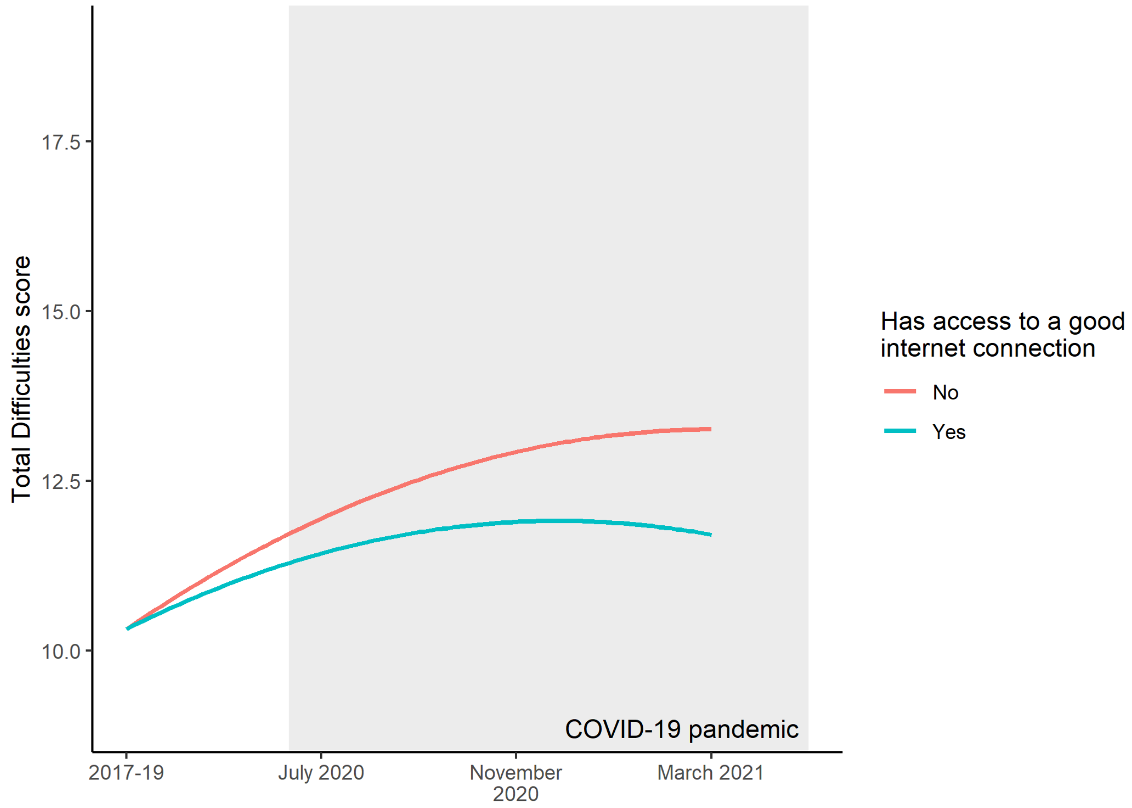

Fig. S2. Sensitivity check: latent growth curve model of youth SDQ Total Difficulties scores, grouped by access to a good internet connection, including sociodemographic control variables and without excluding participants with no longitudinal weight for COVID-19 wave 8.

Table S1. Characteristics of participants with, and without, SDQ Total Difficulties scores at each wave (COVID-19 wave 8 and certain other data suppressed to protect participant identities). When fitting longitudinal models, missing Total Difficulties scores were imputed using full information maximum likelihood estimation.

**Wave**	**All**	**Main study wave 9**		**COVID-19 wave 4**		**COVID-19 wave 6**		**COVID-19 wave 8**	
**SDQ data available**	**All**	**Yes**	**No**	**Yes**	**No**	**Yes**	**No**	**Yes**	**No**
**Total**	1387	638	749	818	569	836	551	1386	1
**Sex**									
Male	656	314	342	372	284	386	270		
Female	731	324	407	446	285	450	281		
Unavailable	0	0	0	0	0	0	0		
**Birth year**									
2004	152	129	23	71	81	68	84		
2005	209	171	38	149	60	137	72		
2006	193	167	26	127	66	124	69		
2007	227	130	97	149	78	153	74		
2008	213	41	172	138	75	134	79		
2009	190	0	190	128	62	129	61		
2010	176	0	176	56	120	91	85		
2011	27	0	27	0	27	0	27		
Unavailable	0	0	0	0	0	0	0		
**Ethnicity**									
White British	973	472	501	590	383	592	381		
Other White	32	19	13	16	16	17	15		
Mixed	135	42	93	81	54	84	51		
Asian	193	87	106	99	94	109	84		
Black	34	15	19	20	14	20	14		
Other									
Unavailable									
**Mean household income (x, annual)**									
x < £20,000	90	54	36	65	25	62	28		
£20,000 ≤ x < £30,000	133	112	21	88	45	93	40		
£30,000 ≤ x < £40,000	155	134	21	106	49	98	57		
£40,000 ≤ x < £50,000	149	124	25	102	47	91	58		
£50,000 ≤ x	258			171	87	167	91		
Unavailable	602			286	316	325	277		

Table S2. Model fit statistics for the ungrouped LGM. Decisions on whether to accept or reject each successive model are made using the p-value from the relevant pairwise likelihood ratio test (LRT).

**Current favoured model**	**Model under test**	**Degrees of freedom (df)**	**Akaike Information Criterion (AIC)**	**Bayesian Information Criterion (BIC)**	χ^2	**LRT *p*-value**	**Significance (* < 0.05 ** < 0.01 *** < 0.001)**
	**No-change**	8	22 101	22 133	182		
**No-change**	**Linear**	5	21 961	22 008	34.7	$< 2.2 \times 10^{-16}$	***
**Linear**	**Quadratic**	1	21 934	22 002	0.16	5.7×10^{-7}	***

Table S3. Ungrouped model output with survey weights applied.

	**Estimate(Std.Err.)**	**p**
**Residual Variances**		
Main study wave 9	10.32(1.85)	< 0.001
COVID-19 wave 4	6.26(0.63)	< 0.001
COVID-19 wave 6	9.03(0.71)	< 0.001
COVID-19 wave 8	0.91(1.92)	0.636
**Latent Intercepts**		
Intercept	10.10(0.15)	< 0.001
Linear coefficient	1.12(0.14)	< 0.001
Quadratic coefficient	-0.26(0.04)	< 0.001
**Latent Variances**		
Intercept	20.72(1.97)	< 0.001
Linear coefficient	7.32(2.29)	0.001
Quadratic coefficient	0.57(0.15)	< 0.001
**Latent Covariances**		
Intercept w/Linear coefficient	-0.10(2.08)	0.962
Intercept w/Quadratic coefficient	-0.42(0.51)	0.405
Linear coefficient w/Quadratic coefficient	-1.69(0.53)	0.001
**Fit Indices**		
χ^2	7.88(1)	0.005
CFI	1	
RMSEA	0.07	

Table S4. Model fit statistics for model grouped by computer access (no covariates). Model 0 is an unconstrained quadratic model. As before, pairwise LRTs are carried out to determine whether the model with an additional parameter constrained to equality (as demarcated) fits significantly worse than the previous model.

**Current favoured model**	**Model under test**	**Additional constraints**	**Degrees of freedom (df)**	**Akaike Information Criterion (AIC)**	**Bayesian Information Criterion (BIC)**	χ^2	**LRT *p*-value**	**Significance (* < 0.05 ** < 0.01 *** < 0.001)**
	**0**		2	15 934	16 057	5.92		
**0**	**1**	Residual variances	6	15 929	16 033	8.88	0.54	
**1**	**2**	*y*-intercept	7	15 928	16 027	9.99	0.29	
**2**	***3***	*Linear coefficient*	*8*	*15 930*	*16 024*	*14.2*	*0.0064*	**
**2**	***4***	*Quadratic coefficient*	*8*	*15 930*	*16 025*	*14.2*	*0.0035*	**
**2**	**5**	*y*-intercept variance	8	15 927	16 021	11.0	0.31	
**5**	**6**	Linear coefficient variance	9	15 925	16 015	11.1	0.89	
**6**	**7**	Quadratic coefficient variance	10	15 924	16 009	11.8	0.40	
**7**	**8**	*y*-intercept/linear coefficient covariance	11	15 922	16 002	12.1	0.57	
**8**	**9**	*y*-intercept/quadratic coefficient covariance	12	15 920	15 996	12.1	0.89	
**9**	***10***	*Linear coefficient/quadratic coefficient covariance*	*13*	*15 920*	*15 991*	*14.3*	*0.041*	*

Table S5. Output for model grouped by computer access (no covariates, survey weights applied).

	With access		Without access	
	**Estimate(Std.Err.)**	**p**	**Estimate(Std.Err.)**	**p**
**Residual Variances**				
Main study wave 9	10.46(2.40)	< 0.001	10.46(2.40)	< 0.001
COVID-19 wave 4	6.27(0.81)	< 0.001	6.27(0.81)	< 0.001
COVID-19 wave 6	9.06(0.92)	< 0.001	9.06(0.92)	< 0.001
COVID-19 wave 8	1.00(2.49)	0.689	1.00(2.49)	0.689
**Latent Intercepts**				
Intercept	10.11(0.19)	< 0.001	10.11(0.19)	< 0.001
Linear coefficient	0.98(0.19)	< 0.001	3.05(0.69)	< 0.001
Quadratic coefficient	-0.22(0.05)	< 0.001	-0.88(0.20)	< 0.001
**Latent Variances**				
Intercept	20.76(2.55)	< 0.001	20.76(2.55)	< 0.001
Linear coefficient	7.07(2.96)	0.017	7.07(2.96)	0.017
Quadratic coefficient	0.53(0.19)	0.005	0.53(0.19)	0.005
**Latent Covariances**				
Intercept w/Linear coefficient	-0.23(2.70)	0.932	-0.23(2.70)	0.932
Intercept w/Quadratic coefficient	-0.37(0.66)	0.57	-0.37(0.66)	0.57
Linear coefficient w/Quadratic coefficient	-1.61(0.69)	0.019	-1.43(0.70)	0.041
**Fit Indices**				
χ^2	39.42(12)	< 0.001		
CFI	0.99			
RMSEA	0.07			

Table S6. Model fit statistics for model grouped by computer access (with covariates). Here the processes as in tables S2 and S4 are combined.

**Current favoured model**	**Model under test**	**Additional constraints**	**Degrees of freedom (df)**	**Akaike Information Criterion (AIC)**	**Bayesian Information Criterion (BIC)**	χ^2	**LRT *p*-value**	**Significance (* < 0.05 ** < 0.01 *** < 0.001)**
	**No-change**		52	17 744	17 915	214		
**No-change**	**Linear**		38	17 650	17 886	91.2	$< 2.2 \times 10^{-16}$	***
**Linear**	**Quadratic (0)**		22	17 618	17 930	27.2	1.0×10^{-6}	***
**0**	**1**	Residual variances	26	17 613	17 906	30.3	0.59	
**1**	**2**	*y*-intercept	27	17 612	17 900	30.8	0.55	
**2**	***3***	*Linear coefficient*	*28*	*17 620*	*17 904*	*41.5*	7.2×10^{-4}	***
**2**	***4***	*Quadratic coefficient*	*28*	*17 621*	*17 905*	*42.7*	*0.0036*	**
**2**	**5**	*y*-intercept variance	28	17 610	17 893	30.9	0.71	
**5**	**6**	Linear coefficient variance	29	17 609	17 888	32.0	0.80	
**6**	**7**	Quadratic coefficient variance	30	17 607	17 882	32.6	0.45	
**7**	**8**	*y*-intercept/linear coefficient covariance	31	17 606	17 876	33.5	0.41	
**8**	**9**	*y*-intercept/quadratic coefficient covariance	32	17 604	17 869	33.6	0.13	
**9**	**10**	Linear coefficient/quadratic coefficient covariance	33	17 603	17 864	34.7	0.29	

Table S7. Output for model grouped by computer access (with covariates, no weights applied).

	With access		Without access	
	**Estimate(Std.Err.)**	**p**	**Estimate(Std.Err.)**	**p**
**Residual Variances**				
Sex	0.25(0.00)	< 0.001	0.21(0.03)	< 0.001
Birth year	0.07(0.00)	< 0.001	0.07(0.01)	< 0.001
Dichotomised ethnicity	0.20(0.01)	< 0.001	0.25(0.00)	< 0.001
Mean monthly household income	0.02(0.00)	< 0.001	0.03(0.01)	0.022
Main study wave 9	8.58(4.05)	0.034	8.58(4.05)	0.034
COVID-19 wave 4	10.21(1.35)	< 0.001	10.21(1.35)	< 0.001
COVID-19 wave 6	8.27(1.35)	< 0.001	8.27(1.35)	< 0.001
COVID-19 wave 8	4.86(3.79)	0.2	4.86(3.79)	0.2
**Latent Intercepts**				
Intercept	9.51(0.78)	< 0.001	9.51(0.78)	< 0.001
Linear coefficient	1.35(0.75)	0.072	10.31(3.37)	0.002
Quadratic coefficient	-0.27(0.20)	0.182	-3.20(1.13)	0.005
**Latent Variances**				
Intercept	21.46(4.39)	< 0.001	21.46(4.39)	< 0.001
Linear coefficient	8.97(5.25)	0.088	8.97(5.25)	0.088
Quadratic coefficient	0.38(0.35)	0.278	0.38(0.35)	0.278
**Latent Covariances**				
Intercept w/Linear coefficient	-2.70(4.45)	0.544	-2.70(4.45)	0.544
Intercept w/Quadratic coefficient	0.27(1.04)	0.796	0.27(1.04)	0.796
Linear coefficient w/Quadratic coefficient	-1.67(1.26)	0.185	-1.67(1.26)	0.185
**Fit Indices**				
χ^2	34.72			
CFI	1			
RMSEA	0.01			
Scaled χ^2	36.05(33)	0.328		

Table S8. Model fit statistics for model grouped by good internet connection access (no covariates). Processes identical to those in Table S6.

**Current favoured model**	**Model under test**	**Additional constraints**	**Degrees of freedom (df)**	**Akaike Information Criterion (AIC)**	**Bayesian Information Criterion (BIC)**	χ^2	**LRT *p*-value**	**Significance (* < 0.05 ** < 0.01 *** < 0.001)**
	**0**		2	15 926	16 048	1.78		
**0**	**1**	Residual variances	6	15 921	16 025	5.51	0.56	
**1**	**2**	*y*-intercept	7	15 920	16 019	5.76	0.61	
**2**	***3***	*Linear coefficient*	*8*	*15 921*	*16 016*	*9.36*	*0.034*	*
**2**	**4**	Quadratic coefficient	8	15 920	16 015	8.39	0.080	
**4**	**5**	*y*-intercept variance	9	15 919	16 009	8.97	0.44	
**5**	**6**	Linear coefficient variance	10	15 917	16 002	8.97	0.99	
**6**	***7***	*Quadratic coefficient variance*	*11*	*15 918*	*15 998*	*11.8*	*0.044*	*
**6**	**8**	*y*-intercept/linear coefficient covariance	11	15 915	15 995	9.18	0.62	
**8**	**9**	*y*-intercept/quadratic coefficient covariance	12	15 915	15 991	11.5	0.12	
**9**	***10***	*Linear coefficient/quadratic coefficient covariance*	*13*	*15 922*	*15 993*	*19.9*	*5.9×10^{-4}*	***

Table S9. Output for model grouped by good internet connection access (no covariates, survey weights applied).

	**With access**		**Without access**	
	**Estimate(Std.Err.)**	**p**	**Estimate(Std.Err.)**	**p**
**Residual Variances**				
Main study wave 9	10.74(2.40)	< 0.001	10.74(2.40)	< 0.001
COVID-19 wave 4	6.32(0.81)	< 0.001	6.32(0.81)	< 0.001
COVID-19 wave 6	8.99(0.92)	< 0.001	8.99(0.92)	< 0.001
COVID-19 wave 8	0.78(2.49)	0.754	0.78(2.49)	0.754
**Latent Intercepts**				
Intercept	10.05(0.19)	< 0.001	10.05(0.19)	< 0.001
Quadratic coefficient	-0.26(0.05)	< 0.001	-0.26(0.05)	< 0.001
Linear coefficient	1.10(0.18)	< 0.001	1.22(0.28)	< 0.001
**Latent Variances**				
Intercept	20.62(2.55)	< 0.001	20.62(2.55)	< 0.001
Linear coefficient	6.82(2.96)	0.021	6.82(2.96)	0.021
Quadratic coefficient	0.56(0.19)	0.003	0.15(0.23)	0.508
**Latent Covariances**				
Intercept w/Linear coefficient	-0.01(2.69)	0.998	-0.01(2.69)	0.998
Intercept w/Quadratic coefficient	-0.44(0.65)	0.5	-0.44(0.65)	0.5
Linear coefficient w/Quadratic coefficient	-1.62(0.69)	0.019	-0.75(0.73)	0.302
**Fit Indices**				
χ^2	49.42(12)	< 0.001		
CFI	0.98			
RMSEA	0.09			

Table S10. Model fit statistics for model grouped by good internet connection access (with covariates). Processes identical to those in tables S6 and S8.

**Current favoured model**	**Model under test**	**Additional constraints**	**Degrees of freedom (df)**	**Akaike Information Criterion (AIC)**	**Bayesian Information Criterion (BIC)**	χ^2	**LRT *p*-value**	**Significance (* < 0.05 ** < 0.01 *** < 0.001)**
	**No-change**		52	17 771	17 941	206		
**No-change**	**Linear**		38	17 672	17 908	78.6	$< 2.2 \times 10^{-16}$	***
**Linear**	**Quadratic (0)**		22	17 652	17 964	26.7	6.1×10^{-5}	***
**0**	**1**	Residual variances	26	17 647	17 940	29.8	0.65	
**1**	**2**	*y*-intercept	27	17 646	17 934	30.8	0.26	
**2**	**3**	Linear coefficient	28	17 645	17 929	31.7	0.33	
**3**	**4**	Quadratic coefficient	29	17 644	17 923	32.6	0.47	
**4**	**5**	*y*-intercept variance	30	17 642	17 917	33.2	0.55	
**5**	**6**	Linear coefficient variance	31	17 640	17 910	33.3	0.78	
**6**	**7**	Quadratic coefficient variance	32	17 642	17 907	36.7	0.062	
**7**	**8**	*y*-intercept/linear coefficient covariance	33	17 640	17 900	36.7	0.99	
**8**	***9***	*y-intercept/quadratic coefficient covariance*	*34*	*17 646*	*17 902*	*45.3*	*0.015*	*
**8**	**10**	Linear coefficient/quadratic coefficient covariance	34	17 641	17 897	40.3	0.22	

Table S11. Output for model grouped by good internet connection access (with covariates, no weights applied).

	**With access**		**Without access**	
	**Estimate(Std.Err.)**	**p**	**Estimate(Std.Err.)**	**p**
**Residual Variances**				
Sex	0.25(0.00)	< 0.001	0.23(0.01)	< 0.001
Birth year	0.07(0.00)	< 0.001	0.06(0.01)	< 0.001
Dichotomised ethnicity	0.20(0.01)	< 0.001	0.22(0.02)	< 0.001
Mean monthly household income	0.02(0.00)	< 0.001	0.02(0.01)	0.028
Main study wave 9	10.08(4.12)	0.014	10.08(4.12)	0.014
COVID-19 wave 4	10.12(1.34)	< 0.001	10.12(1.34)	< 0.001
COVID-19 wave 6	8.47(1.34)	< 0.001	8.47(1.34)	< 0.001
COVID-19 wave 8	5.20(3.80)	0.171	5.20(3.80)	0.171
**Latent Intercepts**				
Intercept	9.72(0.78)	< 0.001	9.72(0.78)	< 0.001
Linear coefficient	1.62(0.74)	0.029	1.62(0.74)	0.029
Quadratic coefficient	-0.38(0.20)	0.063	-0.38(0.20)	0.063
**Latent Variances**				
Intercept	20.51(4.48)	< 0.001	20.51(4.48)	< 0.001
Linear coefficient	7.47(5.45)	0.171	7.47(5.45)	0.171
Quadratic coefficient	0.28(0.37)	0.443	0.28(0.37)	0.443
**Latent Covariances**				
Intercept w/Linear coefficient	-1.56(4.58)	0.733	-1.56(4.58)	0.733
Linear coefficient w/Quadratic coefficient	-1.31(1.32)	0.323	-1.31(1.32)	0.323
Intercept w/Quadratic coefficient	-0.05(1.08)	0.962	0.75(1.15)	0.511
**Fit Indices**				
χ^2	40.29			
CFI	1			
RMSEA	0.02			
Scaled χ^2	37.28(34)	0.321		

Table S12. Sensitivity check: model fit statistics for model grouped by computer access (no covariates, participants without CV8 longitudinal weight NOT excluded). Processes identical to those in tables S6, S8 and S10.

**Current favoured model**	**Model under test**	**Additional constraints**	**Degrees of freedom (df)**	**Akaike Information Criterion (AIC)**	**Bayesian Information Criterion (BIC)**	χ^2	**LRT *p*-value**	**Significance (* < 0.05 ** < 0.01 *** < 0.001)**
	**No-change**		52	26 311	26 500	299		
**No-change**	**Linear**		38	26 177	26 440	137	$< 2.2 \times 10^{-16}$	***
**Linear**	**Quadratic (0)**		22	26 114	26 461	41.9	2.5×10^{-12}	***
**0**	**1**	Residual variances	26	26 110	26 437	46.6	0.32	
**1**	**2**	*y*-intercept	27	26 110	26 431	48.4	0.31	
**2**	***3***	*Linear coefficient*	*28*	*26 115*	*26 431*	*55.5*	*0.0032*	**
**2**	***4***	*Quadratic coefficient*	*28*	*26 115*	*26 431*	*55.7*	*0.0096*	**
**2**	**5**	*y*-intercept variance	28	26 109	26 424	48.7	0.64	
**5**	**6**	Linear coefficient variance	29	26 109	26 419	50.8	0.28	
**6**	**7**	Quadratic coefficient variance	30	26 108	26 413	52.5	0.063	
**7**	**8**	*y*-intercept/linear coefficient covariance	31	26 107	26 407	53.2	0.45	
**8**	**9**	*y*-intercept/quadratic coefficient covariance	32	26 105	26 400	53.3	0.75	
**9**	**10**	Linear coefficient/quadratic coefficient covariance	33	26 104	26 393	53.9	0.12	

Table S13. Output for sensitivity check computer model with survey weights applied.

	**With access**		**Without access**	
	**Estimate(Std.Err.)**	**p**	**Estimate(Std.Err.)**	**p**
**Residual Variances**				
Sex	0.25(0.00)	< 0.001	0.25(0.00)	< 0.001
Birth year	0.09(0.00)	< 0.001	0.10(0.01)	< 0.001
Dichotomised ethnicity	0.22(0.00)	< 0.001	0.25(0.00)	< 0.001
Mean monthly household income	0.02(0.00)	< 0.001	0.02(0.01)	0.015
Main study wave 9	11.05(3.62)	0.002	11.05(3.62)	0.002
COVID-19 wave 4	9.93(1.08)	< 0.001	9.93(1.08)	< 0.001
COVID-19 wave 6	8.28(1.10)	< 0.001	8.28(1.10)	< 0.001
COVID-19 wave 8	4.71(3.44)	0.171	4.71(3.44)	0.171
**Latent Intercepts**				
Intercept	10.20(0.64)	< 0.001	10.20(0.64)	< 0.001
Linear coefficient	1.20(0.64)	0.061	6.33(2.26)	0.005
Quadratic coefficient	-0.22(0.19)	0.225	-2.13(0.86)	0.013
**Latent Variances**				
Intercept	21.87(3.73)	< 0.001	21.87(3.73)	< 0.001
Linear coefficient	7.61(4.63)	0.1	7.61(4.63)	0.1
Quadratic coefficient	0.33(0.33)	0.329	0.33(0.33)	0.329
**Latent Covariances**				
Intercept w/Linear coefficient	-1.39(3.84)	0.718	-1.39(3.84)	0.718
Intercept w/Quadratic coefficient	-0.05(0.93)	0.955	-0.05(0.93)	0.955
Linear coefficient w/Quadratic coefficient	-1.39(1.15)	0.226	-1.39(1.15)	0.226
**Fit Indices**				
χ^2	53.9			
CFI	0.99			
RMSEA	0.03			
Scaled χ^2	53.96(33)	0.012		

Table S14. Sensitivity check: model fit statistics for model grouped by access to a good internet connection (no covariates, participants without CV8 longitudinal weight NOT excluded). Processes identical to those in tables S6, S8, S10 and S12.

**Current favoured model**	**Model under test**	**Additional constraints**	**Degrees of freedom (df)**	**Akaike Information Criterion (AIC)**	**Bayesian Information Criterion (BIC)**	χ^2	**LRT *p*-value**	**Significance (* < 0.05 ** < 0.01 *** < 0.001)**
	**No-change**		52	26 324	26 514	289		
**No-change**	**Linear**		38	26 191	26 454	127	$< 2.2 \times 10^{-16}$	***
**Linear**	**Quadratic (0)**		22	26 133	26 480	37.2	4.3×10^{-11}	***
**0**	**1**	Residual variances	26	26 128	26 455	41.2	0.53	
**1**	**2**	*y*-intercept	27	26 126	26 447	41.2	0.96	
**2**	***3***	*Linear coefficient*	*28*	*26 129*	*26 445*	*45.8*	*0.042*	*
**2**	**4**	Quadratic coefficient	28	26 129	26 445	45.7	0.089	
**4**	**5**	*y*-intercept variance	29	26 127	26 437	45.8	0.74	
**5**	**6**	Linear coefficient variance	30	26 125	26 430	45.8	0.89	
**6**	**7**	Quadratic coefficient variance	31	26 127	26 426	49.2	0.067	
**7**	**8**	*y*-intercept/linear coefficient covariance	32	26 125	26 419	49.6	0.55	
**8**	***9***	*y-intercept/quadratic coefficient covariance*	*33*	*26 130*	*26 419*	*56.5*	*0.039*	*
**8**	**10**	Linear coefficient/quadratic coefficient covariance	33	26 126	26 415	52.4	0.28	

Table S15. Output for sensitivity check internet model with survey weights applied.

	With access		Without access	
	**Estimate(Std.Err.)**	**p**	**Estimate(Std.Err.)**	**p**
**Residual Variances**				
Sex	0.25(0.00)	< 0.001	0.25(0.00)	< 0.001
Birth year	0.09(0.00)	< 0.001	0.08(0.01)	< 0.001
Dichotomised ethnicity	0.22(0.00)	< 0.001	0.23(0.01)	< 0.001
Mean monthly household income	0.02(0.00)	< 0.001	0.02(0.01)	0.003
Main study wave 9	11.69(3.65)	0.001	11.69(3.65)	0.001
COVID-19 wave 4	9.94(1.07)	< 0.001	9.94(1.07)	< 0.001
COVID-19 wave 6	8.30(1.09)	< 0.001	8.30(1.09)	< 0.001
COVID-19 wave 8	5.42(3.47)	0.119	5.42(3.47)	0.119
**Latent Intercepts**				
Intercept	10.32(0.64)	< 0.001	10.32(0.64)	< 0.001
Quadratic coefficient	-0.33(0.18)	0.072	-0.33(0.18)	0.072
Linear coefficient	1.44(0.63)	0.022	1.96(0.85)	0.021
**Latent Variances**				
Intercept	21.40(3.76)	< 0.001	21.40(3.76)	< 0.001
Linear coefficient	7.07(4.72)	0.135	7.07(4.72)	0.135
Quadratic coefficient	0.26(0.35)	0.451	0.26(0.35)	0.451
**Latent Covariances**				
Intercept w/Linear coefficient	-0.94(3.90)	0.809	-0.94(3.90)	0.809
Linear coefficient w/Quadratic coefficient	-1.22(1.18)	0.302	-1.22(1.18)	0.302
Intercept w/Quadratic coefficient	-0.22(0.95)	0.818	0.45(1.02)	0.656
**Fit Indices**				
χ^2	52.44			
CFI	0.99			
RMSEA	0.03			
Scaled χ^2	48.77(33)	0.038		
